# Amputation: A Ten-Year Survey

**DOI:** 10.5812/traumamon.11693

**Published:** 2013-10-14

**Authors:** Amene Sabzi Sarvestani, Afshin Taheri Azam

**Affiliations:** 1 Department of Surgery, Imam Ali Educational Hospital, Zahedan University of Medical Sciences, Zahedan, IR Iran; 2 Department of Orthopedics, Tehran University of Medical Sciences,Tehran, IR Iran

**Keywords:** Amputation, Iran, Etiology

## Abstract

**Background:**

Limb loss occurs due to different causes and has been increased in many countries. It has without exception, great economic, psychological and social impacts.

**Objectives:**

This study assesses the demographics of amputees in one city of Iran.

**Patients and Methods:**

This retrospective study was undertaken on all of the amputees between April 2002 and December 2011. Patients’ demographics including age, sex, the amputated limb, etiology of limb loss and level of amputation were recorded.

**Results:**

We had 216 patients in the study. The average number of amputations was 21.6 per year and varied from 14 to 32. The mean age of amputation was 39.26± 12.6 years. Of the patients, 172 were male (79.62%) and 44 female (20.37%); 119 of the amputations (55.09 %) were major and 97 minor (44.9 %). The most common cause of amputation was trauma and the most common was the toe. In trauma patients the mean age was 38.12± 10.25 years and 98 (83.7%) were male.

**Conclusions:**

In contrast to similar studies in developed countries, trauma was found to be the major cause of all types of amputations. Results of this study may be used in prevention planning.

## 1. Background

Limb amputation is one of the most ancient of all surgical procedures with a history of more than 2500 years dating back to the time of Hippocrates (1). It has many cases such as trauma, peripheral vascular disease, tumor, infection and congenital anomalies (2, 3). Amputation is the last resort when limb salvage is impossible or when the limb is dead or dying, viable but nonfunctional or when it is threatening the patient's life (1). Limb loss often has profound economic, social and psychological effects, especially in developing countries where the prosthetics are poor (4-6). Major limb amputations beside high perioperative mortality are disfiguring (7). In Western countries, rise in amputations is for the most part due to increased life expectancy. According to the newest statistics in the USA, about 1.7 million people live with amputations (8), and the number has increased in the recent years (9). It is estimated that 25–27 in 100,000 of the German population have undergone amputation (10). The incidences of different pathologies leading to limb amputation have been reported to vary in different populations. In developed countries peripheral vascular disease is the major cause; whereas, trauma, infections, uncontrolled diabetes mellitus and malignancies are the leading causes for amputation in developing countries (11, 12). Most amputees in developed countries elderly patients with vascular problems (7, 13-15). However in the developing countries, most patients with amputation are young and the major cause of limb amputation varies from one hospital to another. For patients, knowledge of their health condition or disease plays an important role in improving the quality of life (16).

## 2. Objectives

We studied the epidemiology of amputees in Zahedan, over a 10-year period.

## 3. Patients and Methods

This retrospective chart study was performed by referring at two major teaching and referral hospitals of Zahedan. The records of patients amputated from April 2002 to December 2011were reviewed, and data including age, sex, amputated limb, the level, and the cause of amputation was obtained. 

Amputations were divided into two groups: major, above the wrist on the arm or above the ankle on the leg, and minor, below the wrist or ankle. The main cause of amputation was considered. For qualitative data, statistical evaluations were performed by Chi-square test and quantitative variables were compared between groups using the Student’s t-test. Data was analyzed using SPSS 15 software. A P-value less than 0.05 was considered statistically significant.

## 4. Results

**Figure 1.**
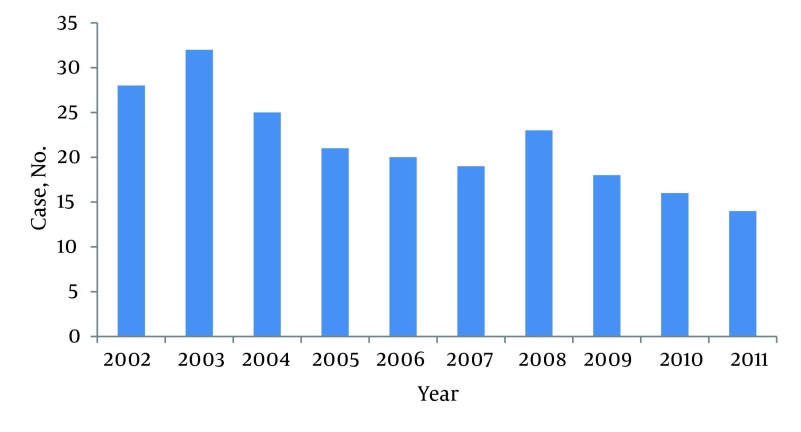
Distribution of Amputation Cases in the Study Period

**Figure 2.**
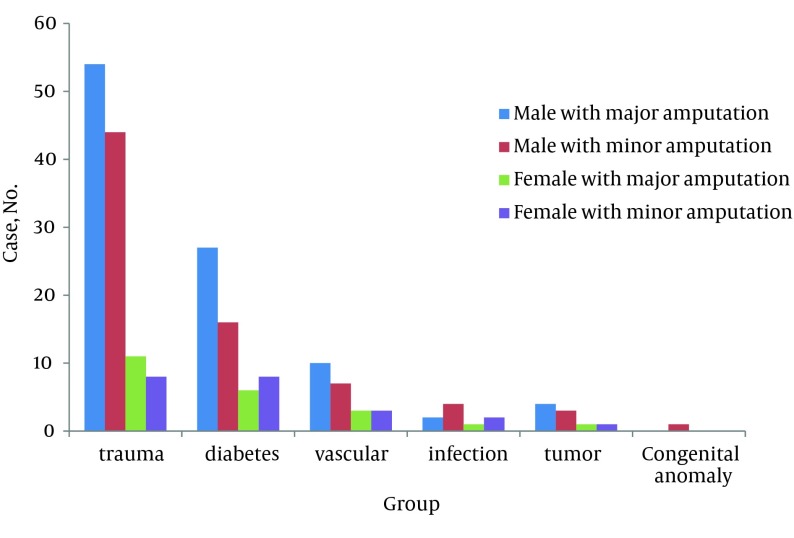
Sex Distribution of Amputation Based on the Etiology

**Figure 3.**
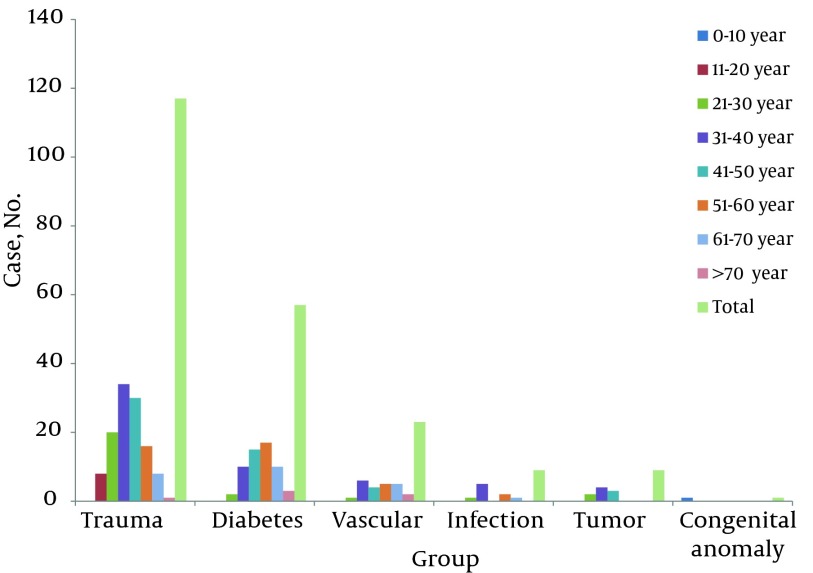
Age Group Distribution of Amputation Based on the Etiology

A total of 216 patients were studied, of whom 198 were amputated for the first time and 18 were amputated more than once. The average number of amputations was 21.6 per year and varied from 14 to 32. Most amputations were performed in 2003 (Figure 1). Patients’ ages varied between 3 and 73 years and, on average, 39.26 ± 12.6 years; 172 patients were male (79.62%) and 44 female (20.37%). Figure 2 shows sex distribution of amputation based on the etiology. The average age of men at amputation was less than that of women; 38.15 ± 12.2 years in comparison to 43.4 ± 14.4 (P = 0.014). Figure 3 shows the age group distribution based on the etiology. 

The lower limb (172 cases or 79.62%) was amputated more than the upper limb (40 cases or 16.51 %). Major amputations comprised 55.09% of all amputation procedures (119 cases), and 44.9% were minor (97 cases). Of all the major amputations, 97 cases (81.51 %) occurred in men, as did 75 cases of minor amputations (77.31 %). The association between two sexes and major and minor amputations was not statistically significant (P = 0.49). 

**Figure 4.**
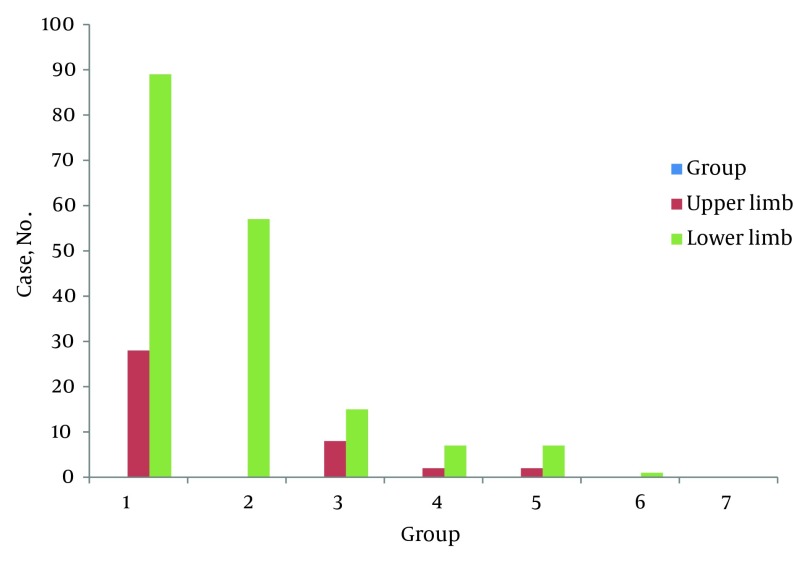
Shows Upper and Lower Limbs Distribution of Amputation Based on the Etiology

Of the upper limb amputations, 62.8 % (49 cases) were major and 37.17 % (29 cases) minor. In the lower limb, 51.44 % of amputations were major (71 cases) and 48.55% minor (67 cases), and this was not statistically significant (P = 0.141). The most common cause of amputations was trauma (117 cases or 54.16%). Diabetes in 57 patients (26.38%) was the second cause of amputation; 23 (10.46%) had severe obstruction of blood vessels with or without gangrene or vascular embolism. The remainder of the amputations were due to infections (osteomyelitis or fasciitis) in 9 (4.1%), soft tissue sarcoma (1.84%), osteogenic sarcoma (1.38%), melanoma (0.46%), squamous cell carcinoma of skin (0.46%), and congenital anomalies (0.46%) (Figure 4).Among the amputations, above-the-knee amputations and toes were more common than others (56 cases (25.92%) and 54 (25%) respectively). Both major and minor amputations were more prevalent in the lower limbs. All of patients with diabetic causes had lower extremity amputations. Of the 18 patients who were amputated more than once, 8 patients were cases of trauma, 6 patients had diabetes, and 4 had vascular diseases. The average age of these patients was 42.15 ± 11.5 years. When trauma was the cause, the mean age of the patients was 38.12 ± 10.25 years (range from 3 to 66); ninety eight (83.7%) were male while 19 (16.23%) were female. The median time from trauma to hospital admission was 48 minutes (range from 5 minutes to 8 hours). Sixty-five cases were results of blunt trauma (59.09%), 30 (27.27%) were from penetrating injuries, and 15 (13.63%) had burns (9 electrical burn and 6 thermal burn) as the cause of amputation. Of all trauma-caused amputations 65 (55.55%) were major and 52 (44.44%) minor. In major amputations 45 (69.23%) were in lower limb and 20 (30.76%) in upper limb, while in minor amputations 44 (84.61%) were in upper limb and 8(15.38%) in lower limb. The toes (36.36%), above knee amputation (36.36%) and fingers (27.27%) were the common levels of amputation. In trauma patients76 (69.09%) had associated injuries. 43 (39.09%) had injuries to other extremities, 34 (30.9%) injuries to head and neck,16 (14.54%) injury to thorax, 9 (8.18%) abdominal injury, and 6 (5.45%) pelvic injury. The median of hospital stay was 3 days, ranging from 1 to 54 days. Of 216 patients 12 (5.55%) died; 3 had vascular cause of amputation, 3 diabetic, and 4 had trauma with other associated injury, and 2 were IV drug abuser with fasciitis. Postoperative complications occurred in 56 (25.92%) of patients. Surgical site infection (SSI) was the most common postoperative complication occurring in 38 (17.59%) of patients. Amputation revisions were done for 18(8.33%) while 16 (7.4%) had wound hematoma, 10 (4.62%) phantom pain, 7 (3.24%) wound dehiscence, and 4 (1.85%) stump gangrene. 

## 5. Discussion

Limb amputation is a common surgical procedure performed by orthopedic, general, vascular and trauma surgeons for therapeutic reasons to save lives; it has profound economic, social and psychological effects (1). Due to the differences in indications and patterns of amputation between different countries and even different cities in a country, this study was performed to describe our experiences on limb amputations in two teaching hospitals and compare the findings with similar studies in other parts of the world. This may help health services recognize, plan and practice preventive strategies.

In our study the males were dominant (79.62%).In another study that was performed in Kerman, another city in Iran, 81.4% of patients were male (16). The male dominance among patients in the present study agrees with the findings by other authors (4, 17).It seems that because the most common cause of amputation in our study and Mousavi's study (16) was trauma which occurs more in males this can explain the greater occurrence of amputation in males. Most of our patients were in the 4th and 5th decades which is comparable with other studies (4, 7, 17),but is in contrast with another study in Ghana which reported high peak age incidence in the 7th decade (18). Other studies reported even lower peak age incidence (19). In the study by Mousvi's et al. most patients were in 3rd and 4th decades of age and the most common cause of amputation, like our study, was trauma. In a study by Moini amputation in trauma patients were in 3rd and 4th decades of age as well (20). Differences in the causes and patterns of amputation which is variable between different countries can explain these age differences .The younger age in our study might be due to trauma which is the most common cause. The number of amputations performed during the 10-year period, except for 1 year (2003), did not demonstrate a sharp increase or decrease. Considering that the population studied increased over the span of years studied, the rate of amputations probably decreased during the study period. On the other hand, the decrease in amputations performed at the teaching hospitals may also have resulted from the construction of several private surgery centers and other governmental hospitals that began operating during the years of the study. In several studies, complications of diabetic foot ulcers were the most common indication for major limb amputation, followed by trauma and peripheral vascular diseases (7, 18, 21). These findings are not in agreement with other studies which reported trauma as the most common indication for limb amputation (17, 20, 22).In our study, like others in Iran (16), found the major cause of limb amputation to be trauma. Tumors and congenital deformities were the causes of only a low percentage of amputations in our study (10 cases (4.62%)). This may be due to scientific progress and the prevention of amputations from these causes, or it may be due to the fact that amputation is reserved for very advanced cases, especially of the upper limb (23). Most of our amputations were performed in the lower limbs like other studies (4, 16). This finding is in agreement with earlier findings that lower extremities are injured more often than the upper extremities and diabetic gangrene is common in the lower extremities than elsewhere on the body (3, 24, 25). All of patients with diabetics had lower extremity amputations in our study. Several studies reported that below knee amputation was the most common procedure performed (4) and some reported transmetatarsal level as the most common level of amputation (16), but other studies reported above knee amputation as the most common procedure performed (17, 26). The complication rate (25.92 %) in our study was lower compared to that of Essoh et al. (39%) (4) and Chalya (33.3%) (27). Surgical site infection was the most common complication like other studies (4, 27). The mortality rate in the present study (5.55 %) is lower than what reported in other studies (4, 17, 27). The reasons for high mortality rate in other studies are diabetic-related complications, wound sepsis and advanced malignancies with metastasis which were found to be common in our study, but in our study mortality was lower because our patients were younger and trauma was the major cause of amputation which occurred in otherwise healthy people. In conclusion, this study showed the epidemiology of limb loss in Iran is mostly due to trauma.
